# Comparison of 3D-printed and laboratory-fabricated Hyrax on stress distribution and displacement of the maxillary complex: a 3D finite element study

**DOI:** 10.1186/s40510-024-00510-w

**Published:** 2024-03-18

**Authors:** Michael Bocklet, Farhad Ahmadi, Timothy Tremont, Loring Ross, Hai Yao, Ildeu Andrade

**Affiliations:** 1 Department of Orthodontics, College of Dental Medicine, Medical University of South Carolina, 173 Ashley Ave, MSC 507, Charleston, SC 29425 USA; 2 Department of Oral Sciences, Medical University of South Carolina, Charleston, SC USA; 3 Clemson-MUSC Joint Bioengineering Program, Department of Bioengineering, Clemson University, Clemson, SC 29634 USA

**Keywords:** Palatal expansion technique, 3D printing, Finite element analysis

## Abstract

**Objective:**

To analyze and compare the effects of a traditional laboratory-fabricated Hyrax expander (T-Hyrax) and two different 3D-printed Hyrax expander models relative to tension points, force distribution, and areas of concentration in the craniofacial complex during maxillary expansion using finite element analysis.

**Materials and methods:**

Three maxillary expanders with similar designs, but various alloys were modeled: a T-Hyrax, a fully printed Hyrax (F-Hyrax), and a hybrid printed Hyrax (H-Hyrax). The stress distributions and magnitude of displacements were assessed with a 5 mm expansion in a symmetrical finite element model. The areas of interest included the teeth, alveolar processes, midpalatal suture, nasal complex, circummaxillary sutures (CS), and the expanders themselves.

**Results:**

The highest stress value (29.2 MPa) was found at the midpalatal suture of the F-Hyrax, while the lowest stress (0.90 MPa) was found at the temporozygomatic suture in the T-Hyrax. On average, the F-Hyrax increased stress at the CS by 24.76% compared with the T-Hyrax and H-Hyrax. The largest displacements were found at the upper incisor (U1) and anterior nasal spine (ANS). The findings indicated an average increase of 12.80% displacement at the CS using the F-Hyrax compared to the T-Hyrax.

**Conclusion:**

The F-Hyrax exerts more stress and displacement on the maxilla than both the T-Hyrax and H-Hyrax, where the weak link appears to be the solder joint.

## Introduction

Rapid maxillary expansion (RME) is used in orthodontics to address maxillary transverse deficiencies, and the goal for correction is separation of the maxillary halves to increase skeletal width [1]. This split is mechanical in nature, and the better the anchorage system, the more the likelihood of successful separation.

However, some known adverse effects of traditional RME are increased axial inclination of anchor teeth, increased inclination of the alveolar processes, a vertical increase in anchor teeth, and inadequate skeletal expansion as a result of anchor teeth tipping [1, 2]. Moreover, greater separation of the midpalatal suture is seen anteriorly than posteriorly [3]. These side effects tend to increase with increasing age and with less teeth incorporated as anchorage.

A previous study has shown that if less dental tipping is desired, with a more linear anteroposterior opening of the midpalatal suture, the expander structure should be more rigid [1]. The lack of ideal rigidity creates centers of rotation in the maxillary bone. Other ideas to minimize side effects include bonding the appliance to the anchor teeth, expanding at an earlier age, incorporating more teeth as anchorage, and using a temporary bone anchorage device (TAD) as opposed to tooth-borne anchors [1, 4–6].

With the advent of dental computer-aided design and computer-aided manufacturing (CAD-CAM) technology, 3D-printed appliances are gaining popularity worldwide. The printed cobalt-chrome (CoCr) superalloy has a higher yield strength and Young’s modulus, which can increase the rigidity of the appliance as a whole [7]. In addition, the digital workflow of the fabrication process allows for efficiency, true customization for each patient, and the ability to incorporate varying numbers of teeth for anchorage [5].

The finite element method (FEM) has been applied to evaluate the different force systems of many orthodontic appliances. This technology enhances understanding of applied force and, by avoiding clinical tests in humans, reduces the ethical dilemma of in vitro testing [8]. FEM can justify the clinical use of 3D-printed expanders by evaluating the stresses and displacement of the appliance since different structures of the maxillary complex can be modeled and evaluated for the impact analysis of any type of applied displacement. Since the 3D-printed expanders promise to be effective in treating transverse maxillary deficiencies, there is a need to verify their benefits and efficiency in relation to craniofacial displacements and stress distribution. Therefore, this FEM study analyzed and compared the effects of a traditional laboratory-fabricated expander (T-Hyrax) and two different variations of a 3D-printed expander, a fully printed (F-Hyrax) and hybrid printed (H-Hyrax), according to stress distribution and displacements in the craniofacial complex during maxillary expansion. The null hypothesis was that there is no difference in the stress distribution or skeletal displacement in the maxillae between the three expanders.

## Material and methods

This study was approved by the Institutional Review Board (Pro00120510). According to the FEM literature, there is no need for sample size calculation and study power in FEM studies [2, 9]. For each test group, one standardized model was constructed at the beginning of the study, and all tests were applied in a standard way to the same group model. The only changes made to each test group were the material properties of each expander.

**Fig. 1:**
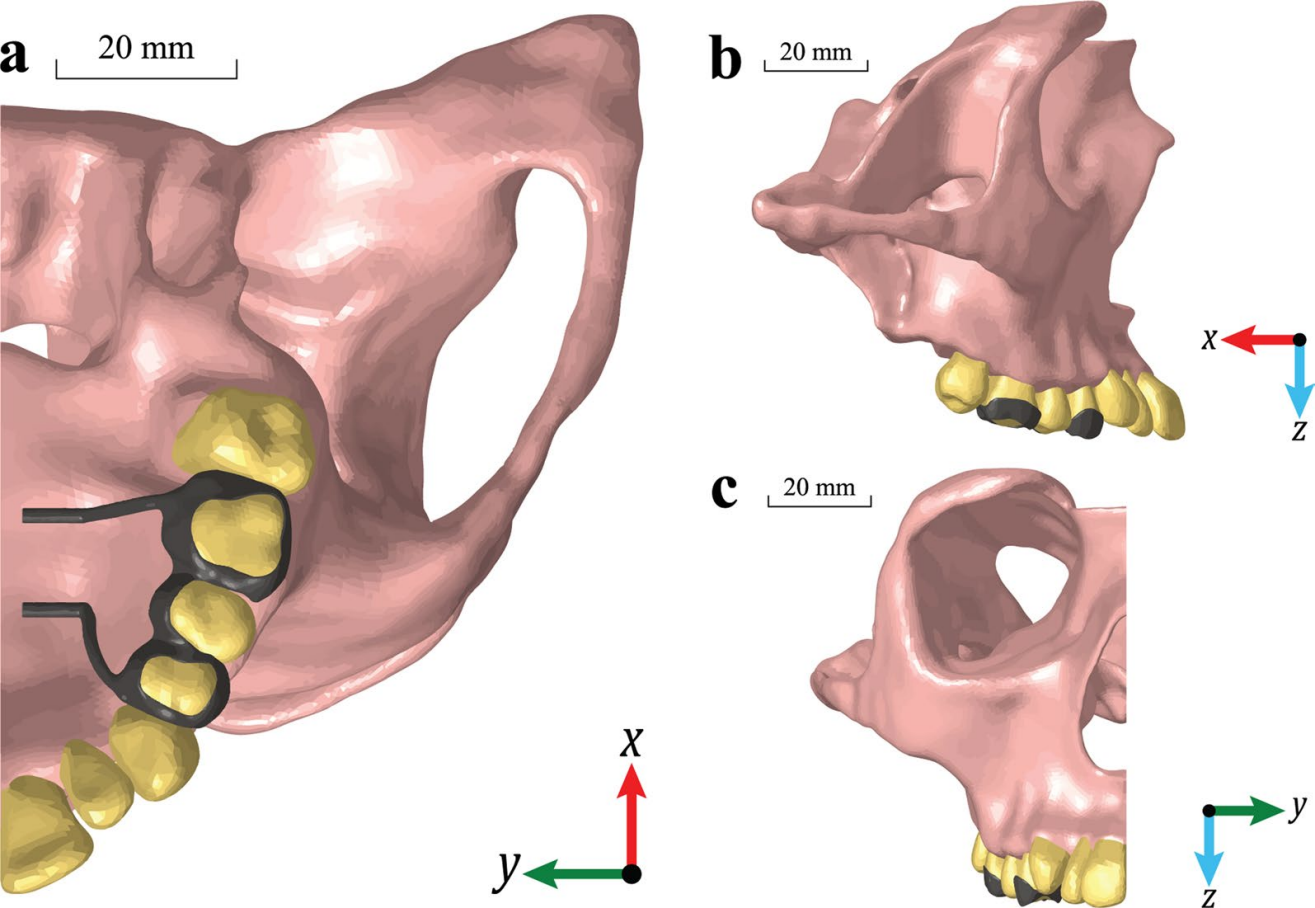
Extent of the geometry and location of the expander**: a** Axial view, **b** Sagittal view, and **c** Coronal view

A FEM model was developed based on the geometry segmented from a cone beam computed tomography (CBCT—0.4 mm voxel size) of a 15-year-old male patient. The segmentation was performed using Amira 6.0.1 (Thermofisher, Waltham, MA). The areas included were the maxilla, circummaxillary sutures (CS), teeth, and the bones that surround the maxilla (Fig. 1). The approximate geometries of the circummaxillary sutures were first segmented out by a practicing orthodontist using Amira and then manually partitioned using Geomagic Wrap, 2021 (Geomagic, Morrisville, NC) by overlaying them onto the maxilla geometry. This process resulted in circummaxillary sutures with varying thicknesses between 1.5 and 2 mm, which were within the range previously reported [10]. The boundaries of the domain for the model geometry were: vertically from Nasion to the upper incisor tip, antero-posteriorly from the upper incisor to the anterior aspect of the foramen magnum (Basion), and transversely from zygion to zygion [2]. The stress and displacement were evaluated in several areas of interest, including teeth (first molar and premolars), maxillary bone, midpalatal suture (anterior, middle, and posterior), CS (zygomaticomaxillary, pterygomaxillary, nasomaxillary, and frontomaxillary), and expander arms and bands.

**Fig. 2:**
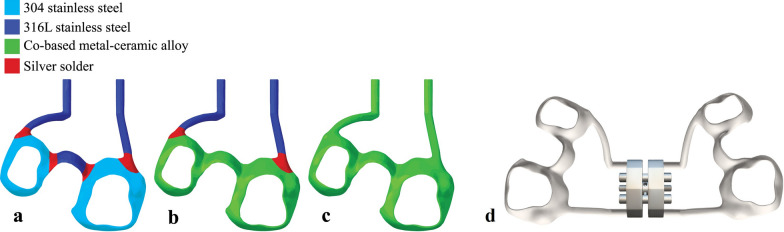
Maxillary Expanders: **a** The T-Hyrax with SS arms and bands soldered together, **b** The H-Hyrax with SS arms soldered to 3D-printed bands, and **c** The F-Hyrax, **d** Appliance design with Hyrax screw

The same geometry (Dean 3 Digital, MN) was used for the three expanders, with the difference in partitioning the solder joints, and the material for the connecting rods, arms, and bands (Fig. 2). The expander geometry was positioned in place via overlaying the patient’s intraoral scan, using the anchorage teeth as the superimposition guides.

Bone, teeth, sutures, and expanders were all considered to be linear-elastic and isotropic materials (Table 1) [10–16]. The loading was of a displacement nature and applied to the expander to resemble the actual process taking place during RME [3, 17, 18]. In each model, a medial–lateral symmetric displacement of 5 mm was imposed on the medial end of the arms, confining the medial end to only have lateral displacement as it is enforced in clinical application by the hyrax screw complex, which is a typical loading condition in FE studies of maxillary expansion [3, 17]. Regarding boundary conditions, all nodes lying on the symmetrical plane were bound to stay in the same plane. Additionally, Basion was completely fixed to prevent any possible rigid body motion [4]. The teeth and the contacting surfaces of the bands were considered to have no relative movement and separation [9]. The model included a total of 691,350 four-node tetrahedral elements with an average maximum edge length of 0.868 mm. Elements, nodes, and mesh sizes were identical for all three models examined in this study (Table 2). The analysis of the developed FEM model was performed using Abaqus/CAE 2018 (Abaqus Inc., Waltham, MA).

**Table 1:** Young’s Modulus and Poisson’s Ratio used in developing the finite element model. Co (Cobalt)

Material	Young’s modulus (MPa)	*P*oisson’s ratio
Maxillary bone [11]	14,900	0.3
Suture [10]	0.67	0.49
Teeth [12]	80,350	0.3
Silver solder [13]	63,400	0.3
316L stainless steel [14]	193,000	0.3
304 stainless steel [15]	180,500	0.3
Co–based metal-ceramic alloy [16]	215,000	0.3

**Table 2:** Mesh metrics for the constructed finite element model

Structure	Avg aspect ratio	Nodes	Elements	Avg min edge length (mm)
Maxilla	1.56	127,321	555,804	0.59
Teeth	1.57	19,923	88,396	0.60
RME	1.69	13,473	47,150	0.26

The values of von Mises stress, a measure of cumulative stresses used to predict yielding and deformation of teeth and bones, were measured in MPa, and displacements were measured in millimeters. Each deformed state and different stress levels were shown using different color-scale bands.

## Results

**Fig. 3:**
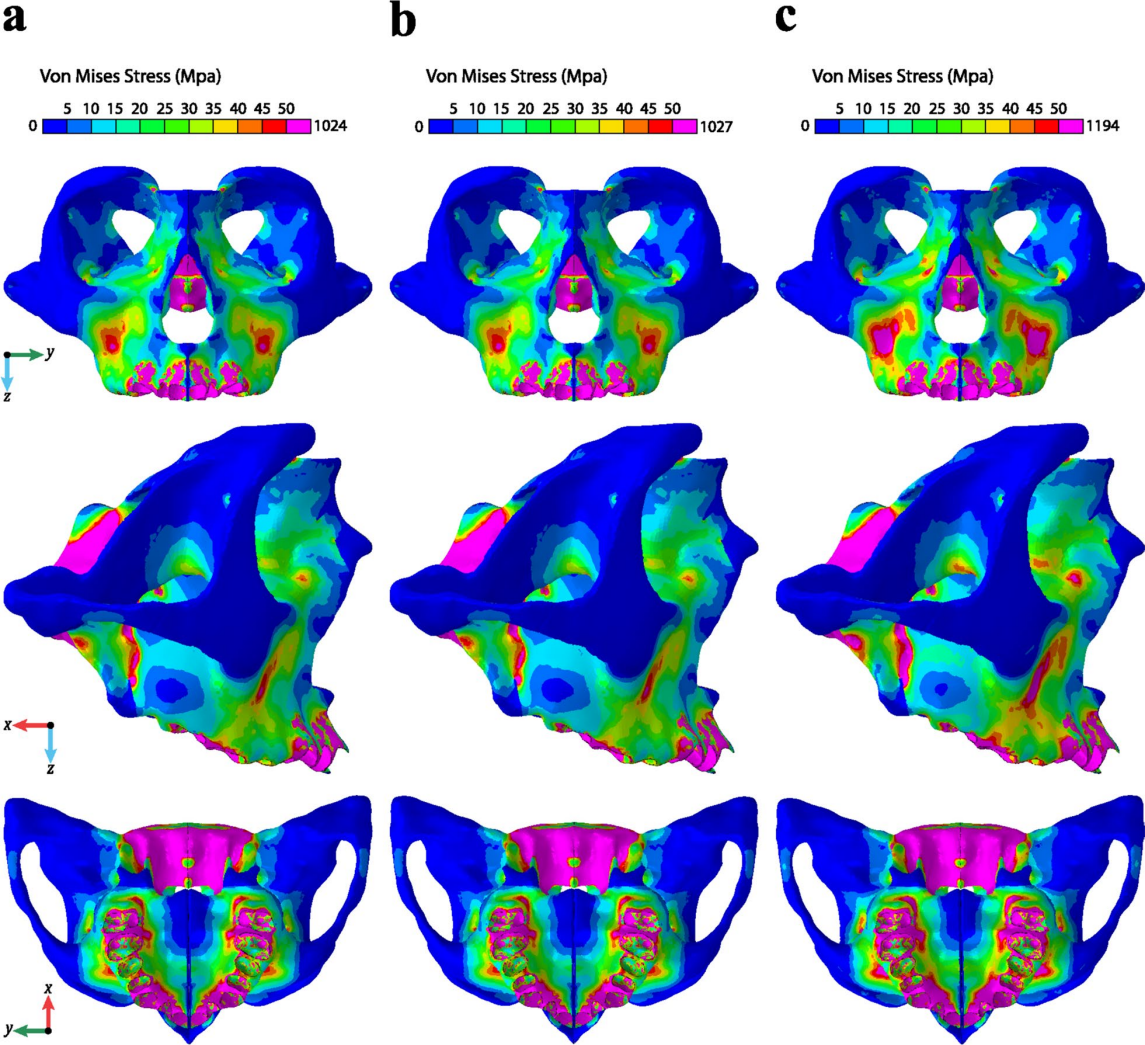
Qualitative stress distribution in the maxilla and part of the surrounding bones: **a** T-Hyrax, **b** H-Hyrax, and **c** F-Hyrax

The highest sutural stress points found with the F-Hyrax were at the midpalatal (29.20 MPa), pterygomaxillary (15.49 MPa), frontonasal (11.90 MPa), and intranasal (4.66 MPa) sutures. The trend demonstrates that the areas of stress concentration within all three models remain the same, but the stress values are markedly larger for the F-Hyrax (Fig. 3). The midpalatal suture (24.42 MPa H-Hyrax/24.35 MPa T-Hyrax) experienced the highest sutural stress values, while the temporozygomatic suture experienced the lowest (1.11 MPa F-Hyrax/0.90 MPa H-Hyrax/0.90 MPa T-Hyrax). On average, F-Hyrax demonstrated increased stress values of the CS by 24.76% compared with T-Hyrax, while H-Hyrax produced only a 0.23% increase over the T-Hyrax.

There was a clear concentration of stress around the sphenoid bone, which accumulated large amounts of von Mises stress in all three models (F-Hyrax 77.66 MPa, H-Hyrax 69.87 MPa, and T-Hyrax 69.83 MPa). The zygomatic buttress and alveolar bone near the anchor teeth also experienced high amounts of stress (Table 3). Similar to the sutural stress evaluations, F-Hyrax delivered the most stress to the maxillary bone and surrounding bony structures, but the pattern of stress distribution (highest to lowest stress areas) was the same for all three models.

**Table 3:** Bony stress evaluations reported as von Mises stress in MPa

Landmark	F-Hyrax	H-Hyrax	T-Hyrax
Medial pterygoid plate	77.66	69.87	69.83
Lateral pterygoid plate	71.50	57.33	57.03
Alveolar bone	25.67	19.39	18.71
Zygomaticomaxillary Buttress	67.31	55.59	55.41

**Fig. 4:**
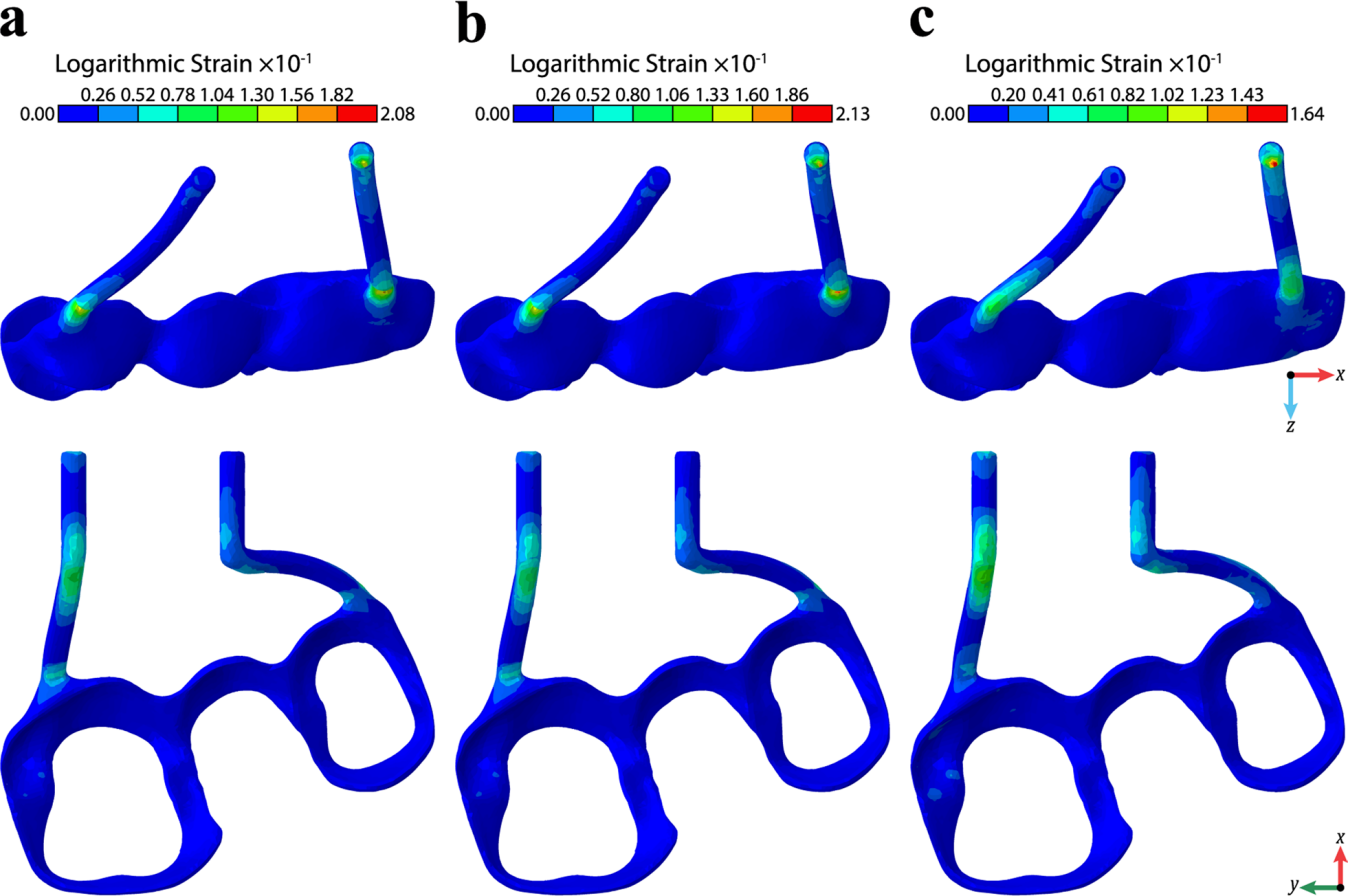
Strain levels in the RMEs: **a** T-Hyrax, **b** H-Hyrax, and **c** F-Hyrax

The T-Hyrax and H-Hyrax expander models experience most of the strain at the level of the solder connection between the bands and RME connector arms, while the F-Hyrax expander model experienced minimal strain at the connection between the RME arms and bands (Fig. 4).

**Fig. 5:**
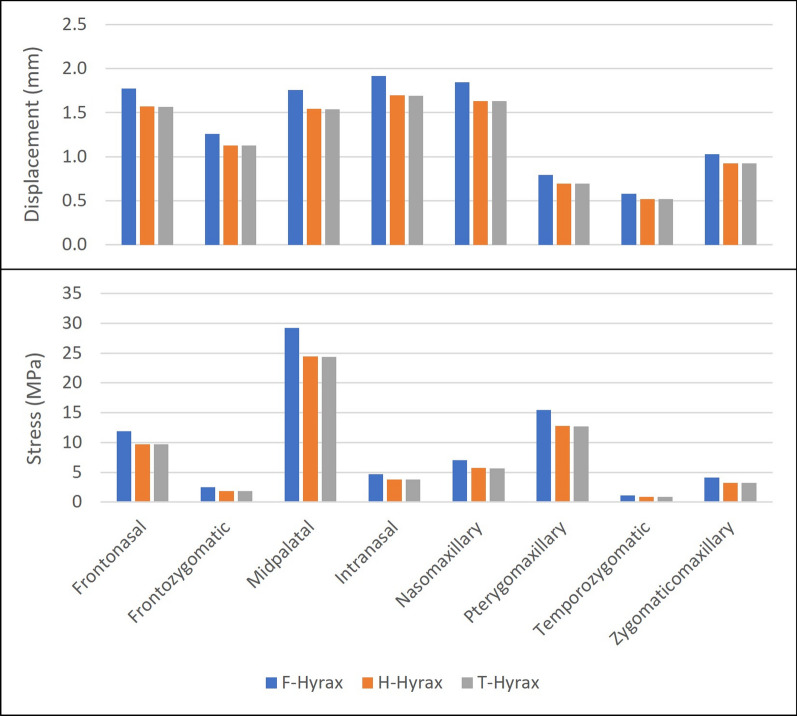
Stress and displacement an average of all the values for elements contacting each CS

As with the stress analysis, the areas of displacement were consistent among all three expander types, showing more displacement anteriorly than posteriorly, but the displacement magnitudes at the sutures were higher in F-Hyrax (Fig. 5). Our findings indicated an average increase of 12.80% displacement at the CS using the F-Hyrax compared to T-Hyrax. The comparison of T-Hyrax vs H-Hyrax demonstrated similar displacement. The average displacement increase for H-Hyrax over T-Hyrax was 0.14%.

**Fig. 6:**
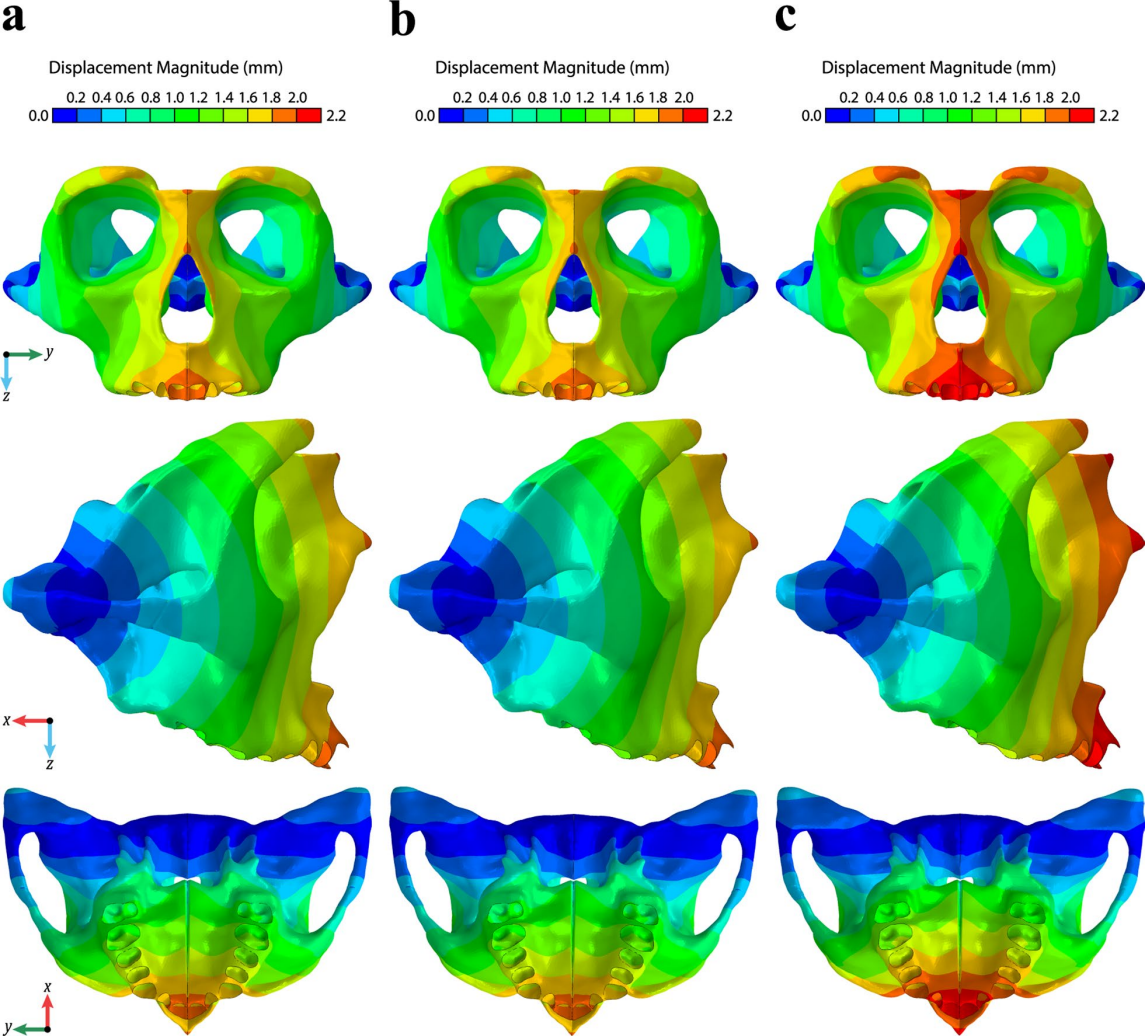
Displacement magnitude in the maxilla and part of the surrounding bones: **a** T-Hyrax, **b** H-Hyrax, and **c** F-Hyrax

In the y-axis (transverse), evaluation of the midpalatal suture displacement showed no predisposition for more linear (parallel) opening within the geometry for the F-Hyrax compared with T-Hyrax or H-Hyrax. The difference between anterior and posterior opening of the suture is 0.2, 0.17, and 0.17 for the F-Hyrax, T-Hyrax, and H-Hyrax, respectively (Table 4). In the z-axis (vertical), evaluation of the displacement showed that all three models demonstrated a downward movement of the maxillary halves along the nodes of the sutures. In the x-axis (anterior posterior direction), the displacement was positive for all models indicating a backward rotation of the nodes within the sutures of interest. The greatest magnitudes of displacement in all three models were seen at the upper incisor (U1), ANS, and upper first premolar (U4) (Fig. 6).

**Table 4:** Coordinate data for points of interest within the maxillofacial skeleton (units = millimeters)

Displacements													
	F-Hyrax					T-Hyrax				H-Hyrax			
**Suture**	Node	x	y	z	Magnitude	x	y	z	Magnitude	x	y	z	Magnitude
Anterior MPS	4,463,047	0.66	0.03	1.69	1.82	0.57	0.03	1.49	1.60	0.57	0.03	1.49	1.60
Middle MPS	4,462,711	0.68	− 0.10	1.24	1.42	0.59	− 0.07	1.09	1.24	0.59	− 0.07	1.09	1.24
Posterior MPS	4,475,646	0.69	− 0.18	0.88	1.13	0.59	− 0.14	0.77	0.98	0.60	− 0.14	0.77	0.99
Midpalatal		0.85	− 0.04	1.51	1.75	0.74	− 0.03	1.34	1.54	0.73	− 0.03	1.33	1.54
Frontonasal		− 0.60	0.00	1.68	1.78	− 0.53	0.00	1.48	1.57	− 0.53	0.00	1.48	1.57
Frontozygomatic		− 0.71	0.08	1.04	1.26	− 0.63	0.07	0.94	1.13	− 0.63	0.07	0.94	1.13
Intranasal		− 0.47	0.00	1.86	1.93	− 0.42	0.00	1.65	1.70	− 0.42	0.00	1.65	1.70
Nasomaxillary		− 0.28	0.02	1.83	1.85	− 0.25	0.02	1.61	1.63	− 0.25	0.02	1.61	1.63
Pterygomaxillary		0.61	− 0.21	0.47	0.80	0.54	− 0.16	0.42	0.70	0.53	− 0.16	0.42	0.70
Temporozygomatic		0.02	− 0.06	0.56	0.57	0.02	− 0.05	0.51	0.52	0.02	− 0.05	0.51	0.51
Zygomaticmaxillary		0.11	− 0.02	1.00	1.01	0.10	− 0.01	0.90	0.91	0.10	− 0.01	0.90	0.91
*Landmark*													
Medial Pterygoid Plate		0.61	− 0.22	0.24	0.69	0.54	− 0.17	0.22	0.60	0.54	− 0.17	0.22	0.60
PNS		0.81	− 0.25	0.73	1.12	0.70	− 0.20	0.64	0.97	0.70	− 0.20	0.64	0.97
U6		1.08	− 0.36	1.03	1.53	0.95	− 0.29	0.92	1.35	0.95	− 0.29	0.92	1.35
U4		1.18	− 0.25	1.49	1.92	1.04	− 0.20	1.32	1.69	1.04	− 0.21	1.32	1.70
U1		1.31	− 0.01	1.99	2.38	1.14	− 0.01	1.75	2.09	1.14	− 0.01	1.76	2.09
ANS		0.70	0.06	1.84	1.98	0.61	0.05	1.62	1.73	0.61	0.05	1.63	1.74

*MPS* Mid-palatal suture. Positive values indicate forward, outward, or upward displacement. Negative values indicate backward, inward, or downward displacement. **x**, x-axis (anteroposterior); **z**, z-axis (longitudinal or vertical); **y**, y-axis (transverse dimensions)

## Discussion

CAD-CAM technology has been a game changer in Orthodontics, and 3D-printed metal appliances are becoming more mainstream in our profession. This was the first FEM study to evaluate whether the higher rigidity of the F-Hyrax could improve the efficacy of skeletal expansion. The results showed the highest amount of stress being delivered to the maxilla and CS using a F-Hyrax as compared to a H-Hyrax or T-Hyrax. In addition, it showed a wedge-shaped expansion with a greater magnitude of displacement in the structures that are positioned more anteriorly on the 3D model.

The increased amount of stress on the CS was accompanied by an increased magnitude of displacement of the maxillary bones with the F-Hyrax. These findings reject the null hypothesis that there is no difference in stress concentration and displacement of the maxilla when comparing the three methods of RME fabrication used in this study. These results support the claims made by Braun et al., that increased rigidity of the appliance can lead to better skeletal expansion by moving the center of rotation higher within the maxilla [1]. The lesser amount of strain in the F-Hyrax arms (Fig. 4) allowed more displacement to be transmitted to the maxillary halves, and biomechanically should result in less dental tipping. In comparison, it is a valid assumption that there is not clinical flex in the connector arms and printed bands of the F-Hyrax. Whereas, the flexibility of the T-Hyrax and the H-Hyrax devices would permit a degree of tooth tipping and therefore less stress and strain associated with the maxillary halves. While this study did not evaluate dental tipping, it clearly showed that the biomechanics demonstrated by Braun hold true, and practitioners can expect a more successful delivery of displacement forces to the maxilla with a more rigid appliance, such as the F-Hyrax.

The stress patterns seen in this study are consistent with other 5 mm-displacement studies, which reported that some of the highest sutural von Mises stress values occurred at the internasal, frontonasal, and nasomaxillary sutures [3, 11]. In addition, there was also a clear trend in the concentration of stress being greatest around the zygomaticomaxillary buttress and pterygomaxillary junction, which is consistent with findings in other studies [1, 19]. Many studies have found that the stress levels located in this area can be very high, causing the pterygoid plates to bend, or in some cases, fracture [20]. This may suggest that careful attention should be given to those patients who are at the point of skeletal maturity when attempting maxillary expansion without surgical assistance. However, the levels of stress (29.20 MPa) seen in the maxilla and CS exceeded those seen in a previous study (17.12 MPa) that compared implant-assisted RME (MARPE) to T-Hyrax [21]. Although not part of this study, our findings indicate that the levels of stress promoted by the F-Hyrax may be comparable to the ones promoted by MARPE. Biomechanically, the high modulus of elasticity of the F-Hyrax likely prevents tipping of the anchor teeth, which may create an equivalent force system that would direct the line of force at a level similar to the that created by the MARPE. Previous 5 mm-displacement RME studies have found higher stress levels to accumulate within their models at the areas of interest, but these studies made the assumption that the expansion device is 100% rigid, which this current study showed to be inaccurate [3, 22]. Moreover, these studies did not model the expander itself, and this study has shown that the T-Hyrax is significantly less efficient at transmitting stress and displacement to the maxilla than the F-Hyrax due to its lack of stiffness at the bands and solder joint. This is the first FEM study to consider different material properties of RME devices and accurately assess the resulting stress and displacement on the maxilla and CS.

The displacement data also reflects that the resistance to expansion was greatest in the posterior portion of the maxilla, with an increased displacement value in the sutures and structures that are positioned more anteriorly on the 3D model (greatest displacements at U1, ANS, and U4). This model does not, however, reflect the trend of increased opening inferiorly in the maxilla as reported by Priyadarshini et al. [23] This may be the result of the limited model size, which excluded the cranial vault, and the high stress levels experienced by the model.

There does not appear to be a significant difference in the stress and displacement values of the H-Hyrax and T-Hyrax. It seems that the solder joint, which is the commonality between both expanders, experienced a high strain, which would lessen the stress delivered to the maxillary bones themselves. Young’s modulus of the silver solder is less than half that of the 316L stainless steel (SS) and CoCr alloy [15, 16]. Thus, this area appears to be a weak point in the design of these two expanders. Some labs may consider welding the expander arms to the printed bands prior to applying silver solder, which may increase the rigidity of this connector site and reduce the bending potential that is present in this area.

Clinically, the T-Hyrax has bands with a connector rod (316L cylindrical piece of SS wire) that connects the molar and premolar bands, and it is adapted to the tooth anatomy. Meanwhile, the H-Hyrax and F-Hyrax models do not extend through the interproximal contact areas and rely on being bonded to the teeth. And their connector arms are continuous with the bands and perfectly adapted to the lingual anatomy of the second premolar. These differences could have made a small impact on this study outcome, but they would also have introduced increased and unwanted variability into it. For that reason, these variables were excluded and can be evaluated in future studies.

Fabrication methods may also impact the outcome. Labs may use different types of SS or different diameters to construct their T-Hyrax or different types of solder material. The degree to which the solder material, or SS, is heated during fabrication can also influence the actual material properties that are present in the appliance after fabrication [24]. Additionally, some labs are laser welding the H-Hyrax bands to the SS expander screw arms [25]. This would certainly increase the appliance’s rigidity as a whole, potentially making the H-Hyrax closer to the performance of the F-Hyrax [26]. Nonetheless, CoCr alloys are more rigid than traditional SS used in traditional fabrication and offer the added advantage of being fully customizable for each patient. [5]

There are some limitations in the FEM studies, as all factors that can affect maxillary expansion cannot be included, such as the patient’s age, bone density, maturity of the midpalatal suture, muscular tonicity, dental tipping, and alveolar bending. Moreover, the different fabrication processes of printed expanders and the expander screw itself were not evaluated. Furthermore, this study did not evaluate the interplay of biology with the mechanics applied during RME, as would be the case with time-dependent FEM models that take those effects into consideration, which could shed light on the way that the teeth and bone respond to the applied forces. The primary concern of this study was to evaluate the efficiency of each appliance in delivering force to the areas of the CS and the relative displacement that resulted from each expander.

## Conclusions

1. The fully printed expander exerts more stress on the maxilla upon displacement than both the hybrid and the traditional devices and therefore also demonstrates more displacement at the sutures of interest.
2. The weak link in both the hybrid and traditional expanders appears to be the solder joint,

making these two appliances almost identical in terms of stress and displacement patterns.

## Data Availability

The authors make the data from this study accessible upon reasonable request.

## Abbreviations

T-Hyrax: Laboratory-fabricated Hyrax expander
F-Hyrax: Fully printed Hyrax expander
H-Hyrax: Hybrid printed Hyrax
CS: Circummaxillary sutures
U1: Upper incisor
U4: Upper first premolar
ANS: Anterior nasal spine
RME: Rapid maxillary expansion
TAD: Temporary bone anchorage device
CAD-CAM: Computer-aided design and computer-aided manufacturing
CoCr: Cobalt-chrome
FEM: Finite element method
CBCT: Cone beam computed tomography
SS: Stainless steel
MARPE: Implant-assisted RME
